# Legislation in 1896

**Published:** 1896-03

**Authors:** 


### LEGISLATION IN 1896.

THIS year should prove a fruitful one in the history of successful legislation of great importance to the veterinary profession, and it becomes the duty of veterinary journalism to keep the profession fully aware of all the different movements and advised, so far as possible, of the merits of these proposed statutes, national, State, and local. To do this we must ask at the hands of every State and local organization that we be kept posted in advance of all proposed legislation, and, so far as possible, by individual veterinarians of any acts pending before legislative bodies in which the veterinarian has a direct or indirect interest. Our columns will be freely opened for the discussion of the merits of these various acts, while our editoral pages will be outspoken in judgment of the value of the same. The JOURNAL has but one chief purpose for its aim, as all its readers must well know by this time, and that is the true advancement in every direction of the profession we represent. Our watchword, to "lead Veterinary Journalism in America," as in the past, will not allow us to spare time or money to fully sustain our worth and influence, and our reward will be in the hope that we have discharged well the serious responsibilities that journalism imposes.

To-day every veterinarian should give his support and exhibit his interest in three Acts now pending before Congress, one of which aims to give rank to veterinarians in the army, and is ably championed by the Army Legislative Committee of the U. S. V. M. A.; a second, Senate Bill 538, introduced by Mr. Sherman, looks to increasing the army and enlarging the number of veterinarians accordingly, with rank designated them; a third, looking to the establishment of a board of registration and examiners for the District of Columbia, should also become a law, and the one point at variance among the veterinarians, as to the composition of the board, should be yielded rather than endanger the passage of the Act.

In New York State some mischievous legislation needs to be looked after and effectually killed. It begins to look as if these repeated efforts to open the doors for registration, save through the channels now properly lodged in the State Board of Examiners, had for its aim some sinister purpose. The correction of the Jury Duty Bill, that its provisions may be enjoyed equally by New York and Brooklyn, should be insured of successful passage.

The proposed plans to enlarge the powers of the State Board of Health in both controlling bovine tuberculosis and extending their aid to local boards, and insuring to the people of the Empire State greater freedom from the dangers of an impure and unwholesome milk- and meat-supply, should be given by every veterinary organization and member of the profession the most earnest consideration. For if greater relief is not afforded by the legislature, *dernier* local measures will be invoked by many of the cities which will work great hardship on the agricultural interests, whose prosperity and protection the veterinarian must sustain and encourage, for his own interests lie largely in the same broad field.

In Massachusetts some proposed modifications of the regulations governing the Tuberculosis Commission have been prepared, and as they are of interest and importance to the public at large, whose guardian in part the veterinarian has become, they should receive earnest consideration at the hands of our members.

In Iowa important and far-reaching changes in dealing more thoroughly with questions involving the purity of the food supply are now pending, and they merit the support of every veterinarian.

In every State where to-day exists large slaughtering establishments involving a national supervision there should be the most cordial and closest coöperation of local authorities to prevent the placing on the home markets that which has been condemned as unfit to be sent abroad for foreign consumption. Self-preservation, like charity, should begin at home.

Legislation in Illinois, Michigan, Virginia, District of Columbia, and other States is under preparation, and in these days of violent partyism, the worst slavery on earth, vicious legislation seems easier of accomplishment than that which is for the common good, and the spoils-system farms favors and privileges away that prove burdens to our people and prejudicial to their welfare and prosperity.

---

Veterinarians of New Jersey should have the Act recently introduced into their State Senate, establishing a Bureau of Animal Industry, so amended that at least two of such Board should be competent, qualified veterinarians. Such appointments are likely to be more satisfactory when made by the Governor rather than when left to one specially interested in but one side of the question. We make this suggestion without any knowledge of who the present or future President of the Board of Agriculture may be, but as the questions involved in this movement have so direct an importance to the whole people from a health point of view, they should be able to immediately place the responsibility for any failure to receive the great benefits to be derived from such proposed legislation as this.

THE successful prosecution by the Examining Board in Ohio, under the direction of Messrs. Meyer, Gribble, and Howe, for violation of the law regulating the practice in the Buckeye State, will teach a wholesome lesson to impostors and those parasites of the profession who have long fed upon the credulity of an innocent, unsuspecting public. There are other Boards that might learn a lesson here which would end the farce that their act threatens to become if a more wholesome respect is not soon taught for its provisions.

